# Laser-Based Length-Measuring Board for the Measurement of Infant Body Length from Outside an Incubator: Proposal and Assessment of a Model

**DOI:** 10.3390/children11121544

**Published:** 2024-12-19

**Authors:** Luís Pereira-da-Silva, Rafael B. Henriques, Daniel Virella, Andreia Mascarenhas, Ana Luísa Papoila, Marta Alves, Horácio Fernandes

**Affiliations:** 1 Neonatology Unit, Hospital Dona Estefânia and Maternidade Dr. Alfredo da Costa, Unidade de Saúde Local São José, Centro Clínico Académico de Lisboa, 1169-045 Lisbon, Portugal; danielvirella@ulssjose.min-saude.pt (D.V.); amascarenhas22@gmail.com (A.M.); 2 Medicine of Woman, Childhood and Adolescence Academic Area, NOVA Medical School, Faculdade de Ciências Médicas, Universidade Nova de Lisboa, 1169-056 Lisbon, Portugal; 3 CHRC—Comprehensive Health Research Centre, Nutrition Group, NOVA Medical School, Faculdade de Ciências Médicas, 1169-056 Lisbon, Portugal; 4 Instituto de Plasmas e Fusão Nuclear, Instituto Superior Técnico, Universidade de Lisboa, 1049-001 Lisbon, Portugal; rhenriques@ipfn.tecnico.ulisboa.pt (R.B.H.); hf@ipfn.tecnico.ulisboa.pt (H.F.); 5 Research Unit, Unidade de Saúde Local São José, Centro Clínico Académico de Lisboa, 1169-045 Lisbon, Portugal; ana.papoila@nms.unl.pt (A.L.P.); marta.alves@ulssjose.min-saude.pt (M.A.); 6 Centre of Statistics and Its Applications, University of Lisbon, 1749-016 Lisbon, Portugal; 7 Population Health Statistical Area, NOVA Medical School, Faculdade de Ciências Médicas, Universidade Nova de Lisboa, 1169-056 Lisbon, Portugal

**Keywords:** caliper, contactless measurement, incubator, laser, length-measuring board, preterm infant

## Abstract

Introduction: Opening the incubator side wall to insert a non-sterile length-measuring device carries the risk of microbial contamination and thermal instability for preterm infants. To reduce this inconvenience, a laser-based length-measuring board is proposed to measure body length from outside the incubator. Methods: This device has two laser-line-shaped cursors which can be pointed to opposite ends of a segment to be measured. It is attached to the outer side of one of the incubator’s side walls in such a manner as to ensure that its axis is parallel to the longitudinal axis of the segment. To validate the measurements made with this model, a calibrated caliper consisting of a conventional rigid length-measuring board with a resolution of 0.05 mm was constructed to serve as a reference. Crown–heel length was measured in a sample of 45 infants, including 32 preterm and 13 term infants of corrected gestational age at the time of measurement. Results: Good intra-observer variability was obtained. Near-perfect statistical agreement was found between measurements with both devices, with concordance correlation coefficients of 0.994 (95% CI: 0.990; 0.996) in preterm infants and 0.994 (95% CI: 0.988, 0.998) in infants at term. The clinical relevance of the agreement between measurements was assessed by a Bland–Altman plot, and the difference may reach clinical relevance (up to 1 cm) but without evidence of proportional bias. Conclusion: The proposed validated laser-based length-measuring board offers a suitable alternative to conventional length-measuring boards for contactless measurement of infant body length.

## 1. Introduction

Body length measurement should be part of the appropriate routine assessment of the nutritional status of neonates in the NICU [1,2]. It should be monitored weekly, and an incremental gain in length of approximately 0.9–1.1 cm/week should be expected [1]. Body length is a more accurate indicator of lean body mass gain than weight because it is not affected by fluid status, making it a better indicator of long-term growth [1,3,4,5]. In preterm infants, linear growth and fat-free mass at discharge are associated with better neurodevelopmental outcomes [6,7,8]. Accurate measurements of body length are particularly important when its value is raised to a power in equations (such as in body mass index) because an inaccurate measurement increases the error of the index in which it is included [9].

Non-flexible length-measuring boards are commonly used to measure the crown–heel length of infants in incubators [1,10]. The use of these devices requires the full opening of a side wall of the incubator to insert a non-sterile device. Once closed, the windows on both sides of the incubator are opened to allow two observers to participate in the length measurement as recommended [6]. Opening the incubator to insert a non-sterile device to measure the body length of preterm infants carries the risk of microbial contamination and infection [11] and thermal instability [12], due to their immaturity.

Lasers are widely used in the medical field and have been shown to be reliable for measuring the length of body segments in humans [13,14].

To mitigate the aforementioned inconvenience caused to preterm infants in incubators, a laser-based length-measuring board is proposed for measurement from outside the incubator. The use of this model eliminates the need to open the entire side wall of the incubator to insert a measuring device and perform a measurement. Therefore, the impact of the measurement is reduced to the opening of the windows on a single side of the incubator, opposite to the side where the device is located, to hold and position the newborn.

The purpose of this paper is to describe the innovative model proposed, how to use it, and to test it by validating its crown–heel length measurements with a precision caliper specifically made to serve as a reference.

## 2. Methods

### 2.1. Conception and Description of the New Device

The proposed device consists of a length-measuring board with laser pointers for the contactless measurement of the crown–heel length and the length of body segments from outside an incubator.

**Figure 1:**
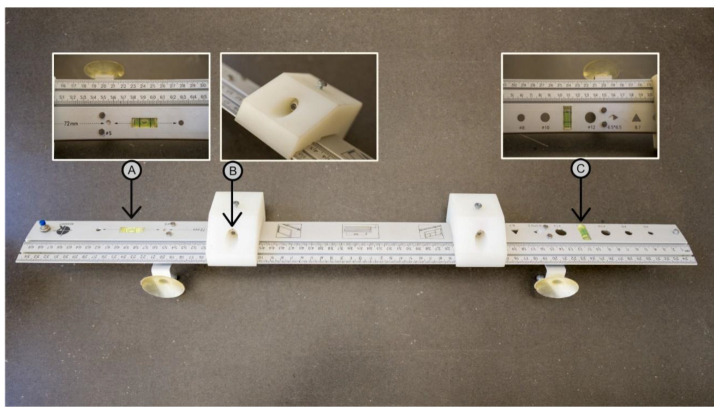
Laser-based length-measuring board: (A) linear spirit level for horizontal alignment; (B) mobile laser support carriage; (C) linear spirit level for vertical alignment.

**Figure 2:**
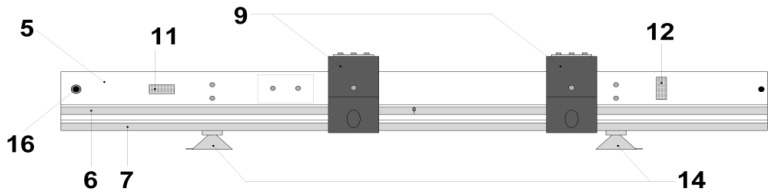
Schematic representation of the laser-based length-measuring board (upper view) including the calibrated axis with two measuring scales, including a calibrated ruler (5) with two-millimeter scales (6 and 7), the mobile laser support carriages (9), the two linear spirit levels (11 and 12), the two vacuum suction cups (14), and the spring switch (16).

**Figure 3:**
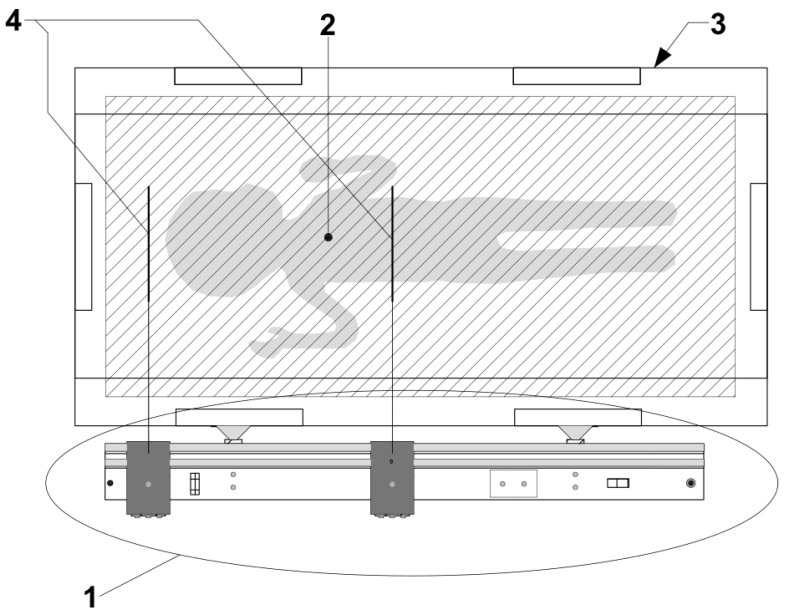
Schematic representation of the laser-based length-measuring board for contactless measurement of body length or body segments from outside an incubator (upper view): the length board (1); the infant (2); the incubator (3); and laser sights (4).

A photo of the proposed laser-based length-measuring board model is presented in Figure 1, and its schematic representation is shown in Figure 2. The most relevant parts of the model are highlighted in more detail in Supplementary Figures S1–S4. A schematic representation (upper view) of this model at the position of operation is shown in Figure 3.

The device (Figure 2) consists of a calibrated axis with two measuring scales, more precisely, a calibrated ruler (5 in Figure 2) with two-millimeter scales (6 and 7 in Figure 2), one of which is symmetrically centered (6 in Figure 2), with the zero point of the scale at the center of the ruler in the longitudinal direction, allowing the length between the laser sights (4 in Figure 3) to be determined simply by summing the two distances indicated on the scale by the moving carriages (9 in Figure 2) when these are in opposite halves of the scale. When the carriages are in the same half of the ruler, for instance, when measuring short lengths (e.g., a body segment), the distance between them is obtained from the subtraction of the highest measured value by the lowest one. The other millimeter scale (7 in Figure 2) begins at one end of the ruler and ends at the opposite end. The ruler (Figure 2) is made of aluminum to ensure the lightness of the entire structure.

On the ruler there are two conductive tracks (17 in Supplementary Figures S1 and S3) made of copper strips. The graphite brushes (8 in Supplementary Figure S3) slide in direct contact with these tracks (17 in Supplementary Figures S1 and S3). These brushes, which are located in the mobile laser carriage (9 in Supplementary Figure S3), allow the laser to be powered (10 in Supplementary Figure S3). In order to avoid gaps between the graphite brushes and the copper tracks, a spring (18 in Supplementary Figure S3) is used, which, through the pressure it exerts on the brush, ensures constant and reliable contact between the track (17 in Supplementary Figure S3) and the brush (8 in Supplementary Figure S3). On the ruler (5 in Figure 2), there are two linear spirit levels (11 and 12 in Figure 2 and Supplementary Figures S1 and S2), which allow the ruler to be precisely placed on a leveled plane, and the transversal leveling screws (13 in Supplementary Figures S1 and S2) can be used to adjust its transversal inclination (5 in Figure 2).

The size of the ruler (5 in Figure 2) determines its use. We have chosen the size of 0.7 m as appropriate for the clinical practice described above.

The reading has an accuracy of half a scale division, i.e., 0.5 mm, although the accuracy can be increased with the introduction of electronic means for determining the distances between the mobile carriages supporting the lasers (9 in Figure 2 and Supplementary Figures S1 and S2) and, consequently, the distances between the targets (4 in Figure 3).

The ruler (5 in Figure 2) is attached to the incubator (3 in Figure 3) or any other similar device using two vacuum suction cups (14 in Figure 2 and Supplementary Figures S1–S3), which can be replaced with a permanent attachment.

The mobile laser carriages (9 in Figure 2 and Supplementary Figures S2 and S3) are made of nylon or another material with a low thermal expansion coefficient and self-lubrication to ensure the best possible orthogonality of the laser (10 in Supplementary Figures S2–S4) or light beam in relation to the calibrated ruler (5 in Figure 2). The maximum deviation allowed with this device is less than half a tenth of a degree (0.05°). This feature also ensures that the light beams are considered parallel to each other along the path between the light source, i.e., the beginning of the light beam, and the crosshair (laser sights, 4 in Figure 3), i.e., the end of the light beam.

The astigmatic lenses (15 in Supplementary Figure S4) applied to the lasers are made of polymethacrylate with a refractive index of 1.4, whose astigmatic dispersion can be adapted to the application and relative orientation of the calibrated ruler (5 in Figure 2) in relation to the premature infant (2 in Figure 3) and the incubator (5 in Figure 3). These lenses (15 in Supplementary Figure S4) are used to diverge a laser beam or any other collimated light beam along an axis, forming thin lines of light whose width is much smaller than their length, thus allowing the extremes of segments to be measured with great precision. The width of the beamline depends on the laser or collimated light source used. The length of the beamline depends on the characteristics of the astigmatic lens, by its radius of curvature, and the distance between the sight and the laser or collimated light source. The application of the instrument will determine the choice of laser or collimated light source and the characteristics of the astigmatic lens.

In the case of the laser sighted length-measuring board (1 in Figure 3), two lines of light are used for measuring (2 in Figure 3). Each of the laser sights (4 in Figure 3) is less than 1.5 mm wide and approximately 60 mm long. Class 2 lasers were used, each with an output power of 1 mW, which is considered safe for the retina given the blink reflex that limits exposure to no more than 0.25 s [15]. Since the laser beam we used is not concentrated but spreads along a line with the above dimensions, the output power will be less than 1 mW if it accidentally hits the eye.

The electrical circuit allows the selection of the viewing time of the sights (4 in Figure 3) via a spring switch (16 in Figure 2 and Supplementary Figures S1–S3) that is normally open, preventing the infant from undue light (2 in Figure 3). The laser supply batteries (19 in Supplementary Figures S1–S3) are coupled to the length-measuring board, thus allowing the lasers (10 in Supplementary Figures S2–S4) to be powered and the length-measuring board to be freely manipulated without the need for an external power supply via a cable.

Since the present invention can be extended to other areas or other conditions of use in neonatology, it is convenient for the light sources to have a variable intensity depending on the application. For this purpose, solid-state lasers or collimated light sources may be available in a wide range of powers to optimize the intensity of the emitted light. The specific requirements of the instrument dictate the power of the light source used. If necessary, the intensity of the light on the sight, i.e., the radiated power, can be regulated more precisely by using an optical intensity attenuator, which typically consists of a thin layer, such as a film with the required percentage of transparency, placed at the output of the laser (10 in Supplementary Figures S2–S4) or collimated light source.

### 2.2. Operation of the Laser-Based Length-Measuring Board

To operate the laser-based length-measuring board for measuring the infant crown–heel length or body segments, the following steps are recommended, schematically represented in Figure 3.

An examiner attaches the device to the outside of the side wall of the incubator with the help of vacuum suction cups. The length-measuring board must be placed parallel to the longitudinal axis of the infant to be measured, so as to provide a measurement as correctly as possible. Since the infant lies in a horizontal plane (the incubator’s plane), the longitudinal linear spirit level of the length board is used to ensure that the length board is placed horizontally.

During measurement, a second examiner is required to open the incubator windows opposite the side where the length-measuring board is located and make sure that the infant remains in the correct position to be measured.

Once the length-measuring board and the infant are secured, one should adjust the length-measuring board’s cross slope using the cross-level adjustment screws. This adjustment should be made so that the laser pointers intersect the distal point of the infant’s body in the longitudinal direction. For this purpose, a switch can be used to temporarily turn on the lasers and check that the pointers correctly intersect the desired points.

When measuring crown–heel length, for added safety, the lasers are turned on far from the eyes of the infant. For absolute safety, a blindfold, identical to that used for phototherapy, can be used, as was performed in this study to test the new model. The laser closest to the eyes should be aimed at the head crown, while the other laser should be aimed at the soles. With the lasers on, the pointers are moved to the desired scan, and then they are turned off. If the cross slope of the length-measuring board is not yet the desired one, the process should be repeated as described above.

For best accuracy, when the pointers are focused on the distal points of the segment to be measured, it should be clear that only half the width of the pointer beamline intersects the specified point. The distance value can then be read directly on the scale of the length-measuring board or, in the case of a digital model, on the axis of the analog or digital display.

### 2.3. Validation of the Laser-Based Length-Measuring Board

#### 2.3.1. Caliper with Adapted Extension Plates

**Figure 4:**
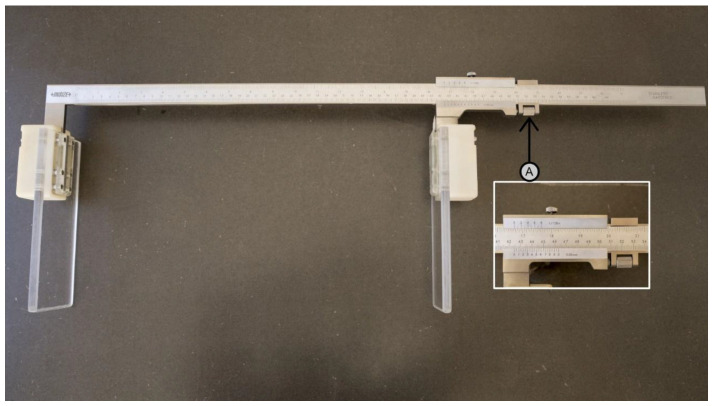
Caliper consisting of a rigid conventional metal length-measuring board, built to validate the laser-based length-measuring board, with a Vernier scale (A) with a resolution of 0.05 mm.

To serve as a reference for validating the measurements made with the laser-based length-measuring board, a calibrated caliper consisting of a rigid metal length-measuring board was purposely adapted for this study. Specifically, it is a solid 600 mm Vernier caliper (model 1208-614; https://insize.com/page-3-71.html, accessed on 18 November 2024) in which two extension plates were adapted, as shown in Figure 4. The plates have a length of 200 mm and a height of 80 mm. Nevertheless, their effective measurement area is reduced to 120 × 80 mm^2^ due to the fixing mechanism that attaches the plates to the fixed and moveable caliper measuring jaws, restricting the effective length of the plate. This length-measuring board contains a Vernier scale with a resolution of 0.05 mm (Figure 4), allowing for reading uncertainty down to 0.025 mm.

#### 2.3.2. Calibration of the Caliper

This caliper is factory calibrated and requires no further calibration. However, it is necessary to check and assess the calibration of the plates that were adapted to the caliper. For this purpose, a calibration standard rod of 125 mm length was used as a reference, as shown in Supplementary Figure S5. The calibration measurements were performed at six distinct positions between the plates as shown in the schematic of Supplementary Figure S6. The readings at these six locations are presented in Supplementary Table S1. The measurement shown in Figure S5 was taken at location C. The results show that the measurement error increases with the distance to the caliper ruler, i.e., toward the end tip of the plates—locations C and F. This behavior is expected because (i) the calibration measurement requires a small amount of force to ensure good contact with the rod and the plates, (ii) the torque increases with the distance from the fixing mechanism, and (iii) the plates have a finite elasticity and therefore allow a slight bending. However, the largest error occurs at location C corresponding to −0.225 ± 0.025 mm. The smallest error on the effective measurement surface is obtained at location E with a deviation of −0.075 ± 0.025 mm. The best results were obtained at points A and D, which are the closest ones to the caliper ruler and where the force momentum is significantly reduced, allowing for deviations of only 0.000 ± 0.025 mm and −0.05 ± 0.025 mm, respectively. The results also show that there is a slight vertical misalignment between the bottom and top of the plates where the locations at the top tend to allow for a 0.025 ± 0.025 mm larger deviation than the locations at the bottom (except for A and D where the difference is 0.05 ± 0.025 mm). To establish a single deviation value for calibration, the average of the four calibration measurements performed in the effective area, B, C, E and F, was calculated. The average deviation value was 124.85 mm. The accuracy achieved was 0.225 mm. It is expected that this accuracy value is a worst-case estimate, as it is likely that when measuring soft body parts, the pressure applied to the measured body/part will be less than during the calibration process, preventing deformation of the measured body/part.

#### 2.3.3. Validation of Measurements Made with the Laser-Based Length-Measuring Board

Measurements made with the laser-based length-measuring board were compared with those made with the caliper used as the reference for validation.

The method used to measure the crown–heel length with the caliper was the same as that recommended using a conventional length-measuring board: with the participation of two examiners, the infant was placed in the supine position; one examiner held the infant’s head against the fixed headboard, aligned with the trunk; the other examiner fully extended the lower limbs by gently pressing down the infant’s knees, holding the feet vertically at right angles to the length-measuring board and moving the footboard up against the heels [9].

The crown–heel length was first measured by one observer (A.M.) using the laser-based length-measuring board and then by another observer (L.P-d-S) using the caliper, who was unaware of the measurements made with the laser-based length-measuring board. In both methods, the crown–heel length value considered was the mean of three consecutive measurements. The examiner using the laser-based length-measuring board was not involved in the technical development of this device.

A convenience sample of 45 infants who were admitted to the neonatal intermediate care unit of the Dr. Alfredo da Costa Maternity Hospital, Unidade Local de Saúde São José, was consecutively recruited, based on the scheduled availability of one of the observers (A.M.) in charge of the validity study. To be included, infants should be clinically stable in intermediate care, not dependent on supplemental oxygen, and receiving exclusive or predominant enteral nutrition at the time of measurement.

In the sample recruited, 20 infants were male, and 11 were twins. As shown in Table 1, six infants were born at term, and 43 were born preterm (less than 37 weeks gestation). At the time of measurement, based on postmenstrual age, the corrected age of 32 children was still preterm, and 13 had reached term age.

The main conditions of the infants were prematurity (*n* = 26), risk of sepsis or sepsis (*n* = 10), jaundice (*n* = 5), intrauterine growth restriction (*n* = 4), hypoglycemia (*n* = 2), and patent ductus arteriosus under conservative care (*n* = 2).

This study was approved by the institutional ethics committee, and the parents of the participants gave informed consent.

##### Comparison and Agreement of Measurements Between the Caliper and the Laser-Based Length-Measuring Board

To compare and validate the crown–heel lengths measured with the laser-based length-measuring board and reference caliper, the following statistical analysis was performed.

To assess the precision of the infant’s body length measurements, intra-observer repeatability was studied using the intraclass correlation coefficient with a corresponding 95% confidence interval. This was estimated using a generalized linear mixed-effects model with a random intercept that considers the correlation structure between repeated measures of the same infant. The study of the agreement between the crown–heel length measurements using the laser-based length measuring board and the caliper as a reference was performed separately for preterm and term infants. To accomplish this, generalized mixed-effects regression models applied to the differences between the two methods were used, as well as the concordance correlation coefficient adapted for repeated measures [16]. This assesses both how close the data are to the line of best fit and how far that line is from the line of perfect agreement (a 45-degree line through the origin). A concordance correlation coefficient of 1 means a perfect agreement. The agreement between the two measurement methods was also assessed by applying a reduced major axis (RMA) regression, used when both independent and dependent variables are measured with error, and by using the Bland–Altman method for repeated measures [17]. The results obtained from applying this method are represented graphically by a Bland–Altman plot, suitable for comparing the two methods from a clinical point of view. A Pearson’s product-moment correlation, with corresponding 95% confidence intervals (95% CI), between the two methods’ differences and corresponding averages was estimated to assess proportional bias (when the agreement between the two methods depends on the observed measurements’ magnitudes).

A level of significance α = 0.05 was considered. Data analysis was performed using Stata (StataCorp. 2023. Stata 18 Base Reference Manual. College Station, TX, USA: Stata Press.) and R software (R: A Language and Environment for Statistical Computing, R Core Team, R Foundation for Statistical Computing, Vienna, Austria, year = 2024, http://www.R-project.org (accessed on 18 November 2024)).

## 3. Results

### 3.1. Intra-Observer Variability

The accuracy of crown–heel length measurements in the total sample was excellent, based on intra-observer repeatability, with an intraclass correlation coefficient of 0.998 (95% CI: 0.997, 0.999) for the caliper measurements and 0.997 (95% CI: 0.995, 0.999) for the laser-based length-measuring board measurements. Similar results were obtained for both methods with preterm and term infants (Supplementary Table S2).

Plots of the two measurement methods against the infants’ identifiers are presented in Supplementary Figures S7 and S8. For both preterm and term infants, a good intra-subject homogeneity of measurements is noticeable. In terms of bias between the two measurement methods, negative and positive differences can be observed.

#### Crown–Heel Length Measurement Comparison

No significant differences were found between the mean crown–heel lengths measured with the caliper and laser-based length-measuring board in either the preterm or term infant groups (Table 2).

### 3.2. Agreement Study

#### 3.2.1. Agreement Analysis in Infants at Preterm Age

The concordance correlation coefficient was almost perfect with an estimate of 0.994 (95% CI: 0.990, 0.996).

**Figure 5:**
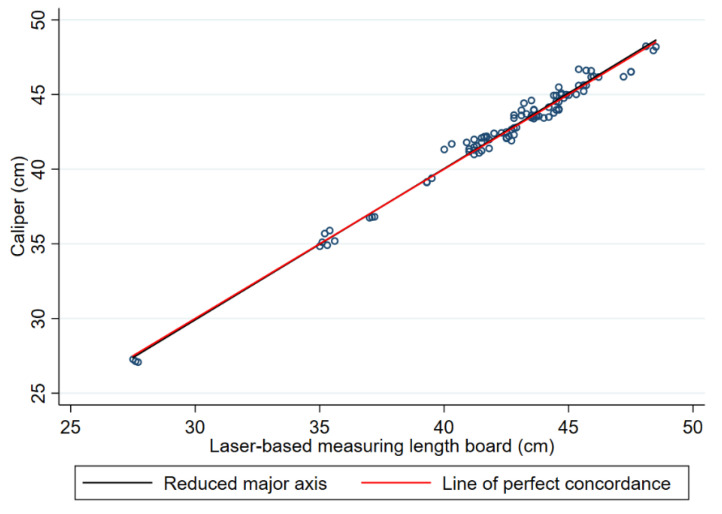
Scatterplot comparing the caliper and laser-based length-measuring board in the crown–heel length measurements of infants at preterm age. Each point corresponds to the individual measurements made on a subject. The black diagonal line is the identity line (x = y). The red line corresponds to the reduced major regression line.

The results of the reduced major axis regression depicted in Figure 5 show that the line of best fit almost overlaps the line of perfect agreement. This result is in line with the high concordance correlation coefficient estimate obtained for infants at preterm age.

**Figure 6:**
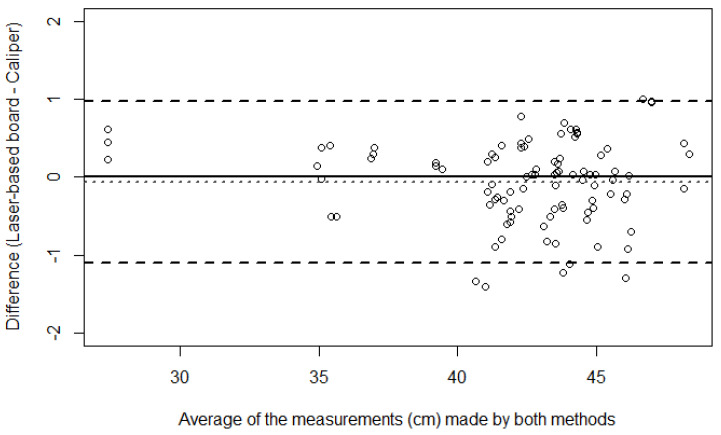
Bland-Altman plot shows the paired differences in cm between the measurement methods against their averages in the crown–heel length measurements of infants at preterm age. Circles correspond to the individual pairs of observations. The dotted line shows the mean bias (−0.061), and dashed lines represent the limits of agreement (upper limit 0.977; lower limit −1.099). The solid black line corresponds to perfect agreement.

Figure 6 shows the agreement by the Bland–Altman method between the caliper and laser-based length-measuring board in the crown–heel length measurements of infants at preterm age.

The Pearson’s correlation coefficient estimate between the two methods’ differences and the corresponding averages was −0.09 (95% CI: −0.29, 0.11; *p* = 0.363), showing no evidence of proportional bias in the Bland–Altman plot.

#### 3.2.2. Agreement Analysis for Infants at Term

The concordance correlation coefficient was almost perfect with an estimate of 0.994 (95% CI: 0.988, 0.998).

**Figure 7:**
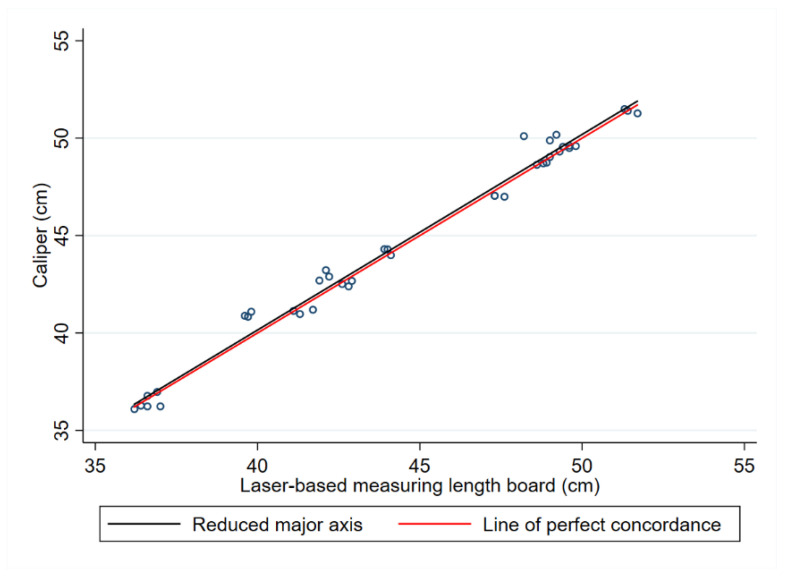
Scatterplot comparing the caliper and laser-based length-measuring board in the crown–heel length measurements of infants at term. Each point corresponds to the individual measurements made on a subject. The black diagonal line is the identity line (x = y). The red line corresponds to the reduced major regression line.

The results of the reduced major axis regression depicted in Figure 7 show that the line of best fit almost overlaps the line of perfect agreement, a similar result obtained for the infants at preterm age. This result is also in line with the high concordance correlation coefficient estimate obtained for infants at term.

**Figure 8:**
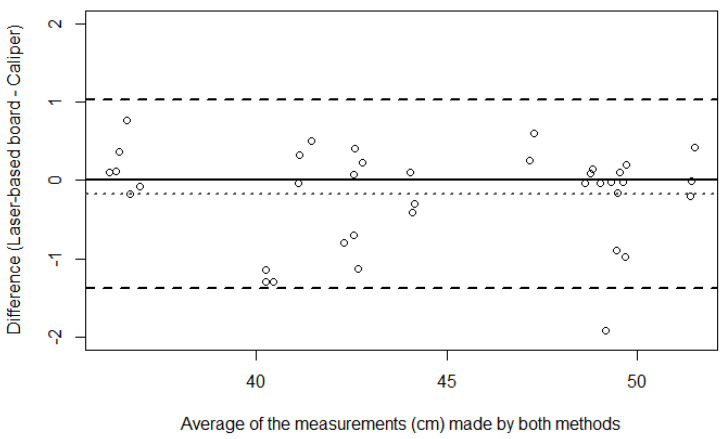
Bland-Altman plot showing the paired differences in cm between the measuring methods against their averages in the crown–heel length measurements of infants at term. Circles correspond to the individual pairs of observations. The dotted line shows the mean bias (−0.170), and dashed lines represent the limits of agreement (upper limit 1.035; lower limit −1.374). The solid black line corresponds to perfect agreement.

Figure 8 shows the agreement by the Bland–Altman method between the caliper and laser-based length-measuring board in the crown–heel length measurements of infants at term.

The Pearson’s correlation coefficient estimate between the two methods’ differences and corresponding averages was −0.03 (95% CI: −0.34, 0.29; *p* = 0.850), showing no evidence of proportional bias in the Bland–Altman plot.

## 4. Discussion

This paper describes a new model of laser-based length-measuring board designed to measure the crown–heel length and body segments of infants from outside an incubator. This device is equipped with laser pointers on each cursor to indicate the ends of the segment being measured, and the measured distance can be read directly on the scale. The measurement can be read on either an analog or digital display in a more user-friendly manner if a similar digital model is developed.

The crown–heel length measurement is essential for monitoring growth and nutritional status in infants, and accurate measurement requires an appropriate measurement technique, equipment, experience, and subject cooperation [6,10,18].

When measuring the crown–heel length of preterm infants in an incubator, inaccuracies have been described when measurements are oversimplified by using tape measures [9,19]. Conversely, the prematometer [20] was developed as a device to improve the accuracy of the crown–heel length measurement of infants in incubators, but its proper use can be cumbersome in routine practice [10].

To reduce the handling of unstable preterm infants, the measurement of body segments, such as knee–heel length [21] and foot length [22], have been proposed as alternatives to crown–heel length, assuming that body segment growth is representative of whole-body growth [23]. More sophisticated contactless body-size-measuring devices, such as 3D techniques, allow stress-free measurement but are time-consuming and not practical for routine use in neonatal intensive care units [24]. Simpler and less expensive length-measuring boards have been commonly used to provide relatively accurate measurements in clinical practice [1,10]. However, introducing any non-sterile measuring device into the incubator has the disadvantage in preterm infants of increasing the risk of contamination and infection due to their immature immune system [11] and thermal instability due to their poor ability to regulate body temperature [12] while the incubator is open for the measurement procedure.

In order to serve as a reference to validate the measurements made with the new laser device, a calibrated caliper consisting of a rigid metal length-measuring board with a Vernier scale with a resolution of 0.05 mm was adapted for the purpose of validation in this study.

An excellent intra-observer repeatability of measurements was found in the total sample, both in infants at preterm and term corrected for gestational age. Good precision was found for crown–heel length measurements using both the caliper and laser-based length-measuring board, again in both preterm and term corrected for gestational age infants.

Considering that lower limb muscle tone may vary according to an infant’s maturity at birth [25] and postnatal age [26], validation of the laser-based crown–heel length measurement was performed separately in preterm and term corrected for gestational age infants. In addition, measuring infants still at a preterm corrected age allowed the assessment of any proportional bias related to the magnitude of length.

No significant differences were found between the mean crown–heel length measured with the caliper and with the laser-based length-measuring board, in either preterm or term corrected for gestational age infants.

A near-perfect statistical agreement was found between crown–heel length measurements using the caliper and the laser-based length-measuring board in preterm and term infants. However, from a clinical point of view, the Bland–Altman graphical analysis showed that the agreement between the measurements made by the two methods is not outstanding, since the difference in measurements in a few individuals may reach clinical relevance (up to about 1 cm), both in preterm and term-corrected infants, but without evidence of proportional bias, i.e., not related to the magnitude of the infant’s length itself.

Comparing both measurement methods, the mean difference between them is negative, which means that in many cases, the caliper readings were longer than those measured with the laser-based length-measuring board. It would be expected that the body length values measured with the laser-based length-measuring board would be longer than those measured with the caliper, since there is some compression of tissues when using the caliper, which does not occur with the contactless measurement method. For best accuracy, when using the laser-based length-measuring board, it is recommended that only half the width of the pointer beamline intersects the points of interest at the ends of the measured segment. We speculate that in the difficulty of easily visualizing each pointer beamline, the observer may have included its full width (up to 1.5 mm) or a large portion of the width at each end of the crown–heel measurement (either the head crown or the soles), resulting in a shorter length than if the recommended technique had been followed to the letter. This suggests that a study should be conducted on the learning curve for using the laser-based length-measuring board.

Two limitations should be acknowledged. First, a convenience sample was used because it was considered unfeasible to recruit using a probabilistic sampling technique. Second, for the validation study of measurements made with the laser-based length-measuring board, only intra-observer variability was assessed. The authors felt that it was not ethical to subject each infant to multiple manipulations by different observers for repeated measurements to assess inter-observer variability with both devices. Additional bias was avoided by ensuring that the examiners of both devices were blinded to each other’s measurements, and none of the examiners who took part in the body length measurements were involved in the technical development of any of the measuring devices.

The new device was designed taking into account the risks expected in clinical practice such as the inevitable decrease in ambient temperature inside the incubator when the side wall is fully opened to insert an object, as well as the risk of contamination if this object is not sterilized and comes into direct contact with the vulnerable newborn. Future studies are needed to assess the extent to which the temperature decreases with this procedure and whether microbial contamination or infection occurs depending on the degree of contamination of the measuring object and devices inserted into the newborn’s environment, including central-line-associated sepsis. It will also be necessary to evaluate whether the harmless output power of the laser beam used eliminates the need for blindfolds when measuring neonatal length in clinical practice.

## 5. Conclusions

A new device for measuring the body length and body segments of preterm infants from outside an incubator is proposed, with the purpose of decreasing the risks of microbial contamination and unintentional reduction in ambient temperature inside the incubator resulting from the opening of the incubator side wall to insert a non-sterile measuring device. The new model is a length-measuring board with both cursors fitted with laser pointers to indicate the references for crown–heel measurement from outside the incubator.

No significant differences were found between the mean crown–heel lengths measured with the reference caliper and the laser-based length-measuring board, either in the preterm or term-corrected infants at the time of measurement. A near-perfect statistical agreement was found between the measurements made with both devices, although the difference in measurements in a few individuals may reach clinical relevance.

The proposed validated laser-based length-measuring board may be a suitable alternative to conventional length-measuring boards to measure infant body length from outside an incubator. Developing a digital version of this model could further improve its usability by providing more user-friendly, real-time readings on an analog or digital display.

## 6. Patent

The invention has been registered as National Utility Model (patent family) N° 10967 at the National Institute of Industrial Property in Portugal (https://inpi.justica.gov.pt/ (accessed on 18 November 2024)).

## Figures and Tables

**Table 1:** Characteristics of the infants (*n* = 45).

Variable	Descriptive Measures
Gestational age at birth (weeks), mean (SD)	31.82 (3.94)
Birth weight (g), mean (SD)	1675.6 (766.2)
Postmenstrual age (weeks) at the time of measurement, median (P_25_–P_75_)	35.0 (33.7–38.1)

P_25_: 25th percentile; P_27_: 75th percentile; SD: standard deviation.

**Table 2:** Comparison of the crown–heel length measurements of infants at preterm and term postmenstrual age, measured with the laser-based length-measuring board and the caliper as reference.

Postmenstrual Age at the Time of Measurement	Gestational Age at Birth (Weeks), Median (P_25_–P_75_)	Postmenstrual Age (Weeks), Median (P_25_–P_75_)	Laser-Based Model, Length (cm)	Caliper, Length (cm) †	*p*-Value
Infants at preterm age (*n* = 32)	31.0 (30.0–33.8)	34.0 (33.3–35.5)	42.3 (4.0)	42.4 (4.1)	0.474
Infants at term (*n* = 13)	36.0 (28.0–39.0)	41.0 (38.9–42.1)	44.5 (5.1)	44.7 (5.1)	0.265

† Ignoring repeated measurements; *p*-values were obtained by mixed-effects regression models to compare both methods of crown–heel length measurement.

## Data Availability

The raw data supporting the conclusions of this article will be made available by the authors on request.

## Supplementary Materials

The following supporting information can be downloaded at: https://www.mdpi.com/article/10.3390/children11121544/s1, Supplementary Figure S1: The rear view of the model, Supplementary Figure S2: The frontal view of the model, Supplementary Figure S3: Side view of the model, Supplementary Figure S4: Laser with the astigmatic lens, Supplementary Figure S5.: Method used to assess the calibration of the plates, Supplementary Figure S6: Schematic of the extension plates representing the calibration measurement locations, Supplementary Figure S7: Scatterplot of crown–heel lengths measured with the two measurement devices for each infant at preterm age, Supplementary Figure S8: Scatterplot of crown–heel lengths measured with the two measurement devices for each infant at term, Supplementary Table S1: Caliper readings during the calibration measurement process at the six locations using a calibration standard rod, Supplementary Table S2: Intra-observer repeatability for crown–heel length measurements in infants at preterm and term-corrected gestational age.
