# Supplementary material for: Laser-Based Length-Measuring Board for the Measurement of Infant Body Length from Outside an Incubator: Proposal and Assessment of a Model
Source: Children (Basel). 2024 Dec 19;11(12):1544. doi: 10.3390/children11121544 (PMC11727513; doi:10.3390/children11121544)
Supplement: Supplementary file 1 [file children-11-01544-s001.zip › children-3350032-supplementary.pdf]

children

MDPI

SUPPLEMENTARY FIGURES

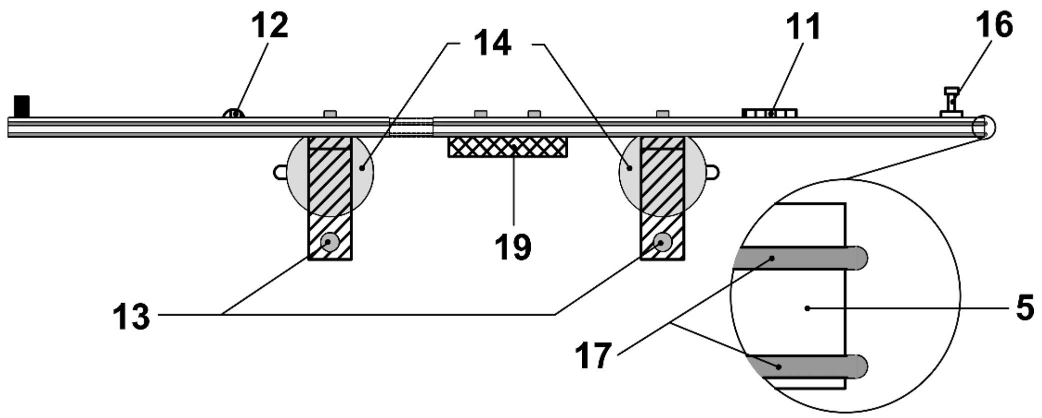

Supplementary Figure S1. The rear view of the model, without the mobile laser support carriages: the axis with the calibrated ruler (5), the two linear spirit levels (11) and (12) that allow the length measuring board to be placed precisely on a plane, the transverse level adjustment screws (13) to adjust the transverse inclination, the two vacuum suction cups (14), the spring switch (16), the two conductive copper tape tracks on the ruler (17) and the laser supply batteries (19).

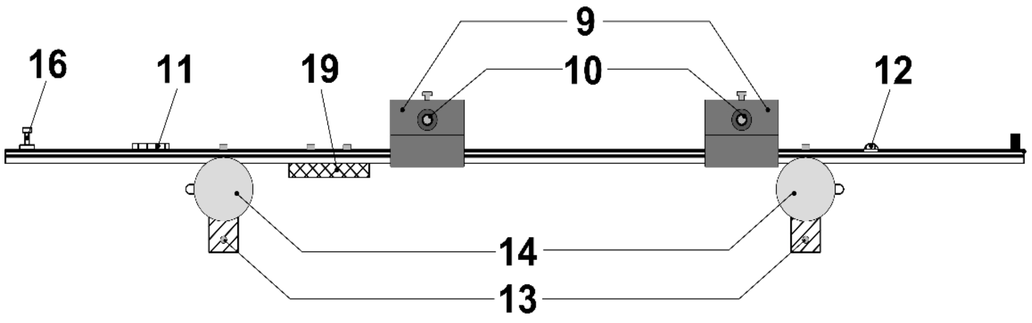

Supplementary Figure S2. The frontal view of the model: the mobile laser support carriages (9), the laser or light beam (10), the two linear spirit levels (11) and (12), the transverse level adjustment screws (13), the two vacuum suction cups (14), the spring switch (16) and the laser supply batteries (19).

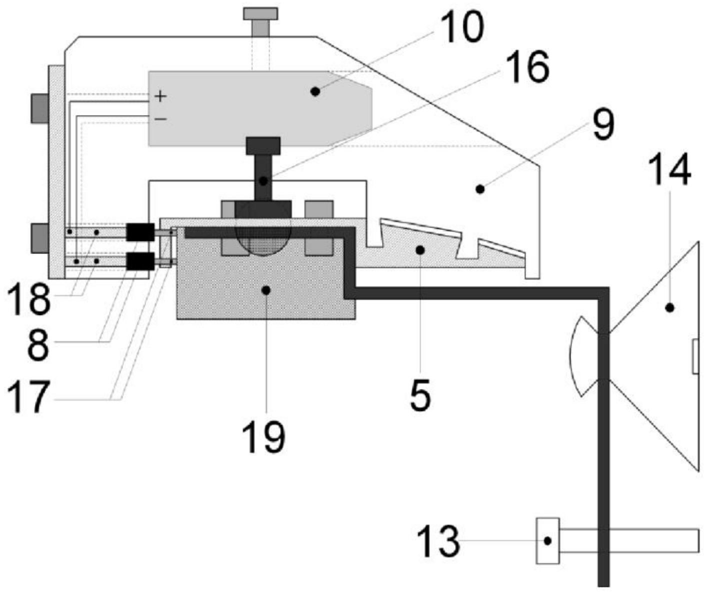

Supplementary Figure S3. Side view of the model, with representation of the elements inside the mobile laser support carriage (9): the axis with the calibrated ruler (5), the graphite brushes (8) which, resident on the mobile laser support carriage (9), allow powering the respective laser or light beam (10), the transverse level adjustment screws (13) to adjust the transverse inclination, one of the vacuum suction cups (14), the spring switch (16), the two conductive copper strip tracks on the ruler (17), the spring (18) that guarantees constant contact between the track (17) and the brush (8), and the supply laser batteries (19).

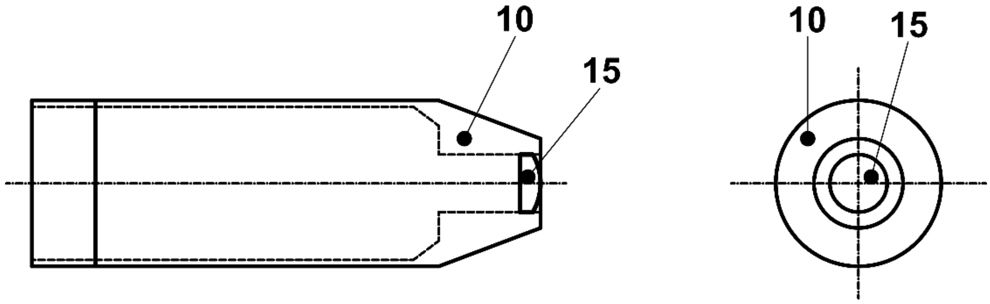

Supplementary Figure S4. Laser (10), with the astigmatic lens (15), seen laterally, on the left, and seen frontally, on the right.

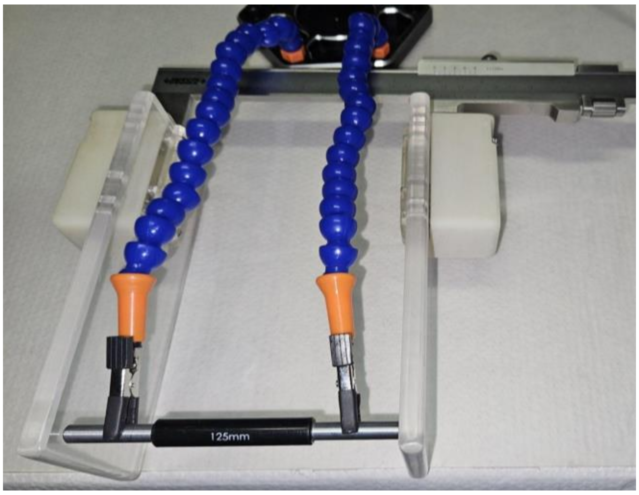

Supplementary Figure S5. Method used to assess the calibration of the plates with a standard 125 mm calibration rod. The location of the measurement shown in this photograph corresponds to the location letter (C) in the schematic of Supplementary Figure S6.

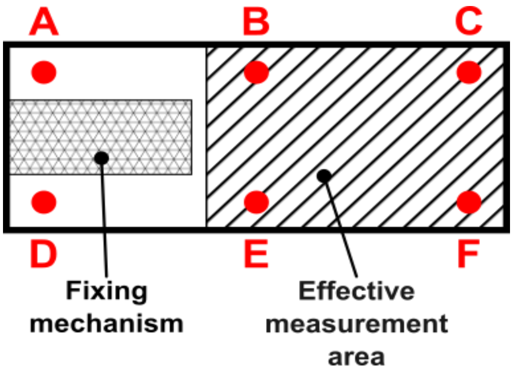

Supplementary Figure S6. Schematic of the extension plates representing the calibration measurement locations (red dots with capital letters). Locations A, B, and C are at the top (the side of the scale on the caliper) and D, E, and F are at the bottom. A and D are closer to the caliper ruler while C and F are at the opposite end (further away from the caliper jaws).

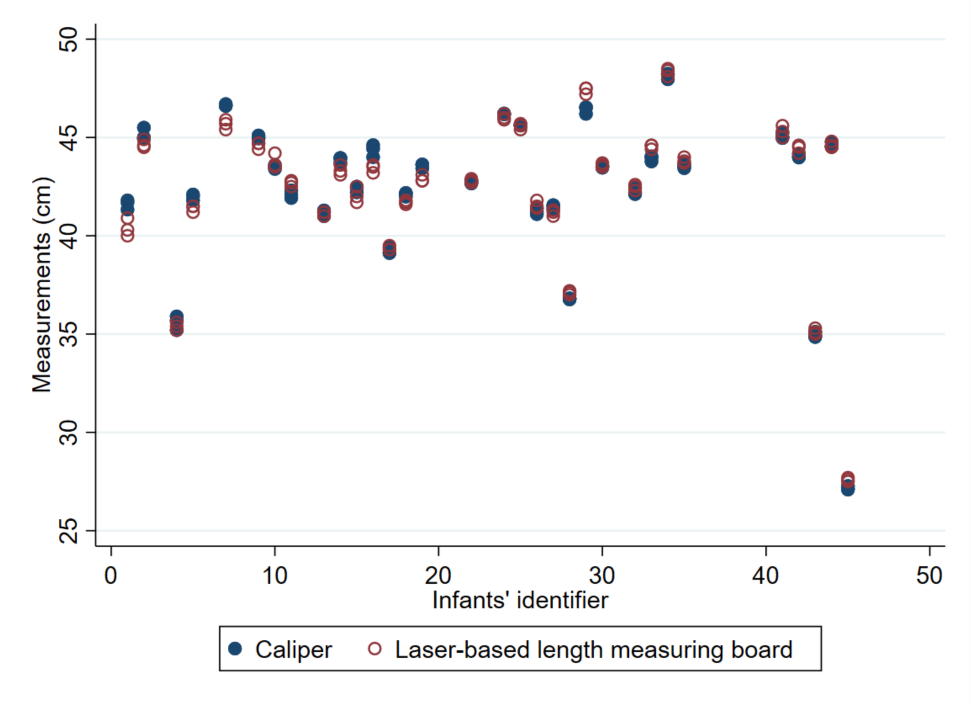

Supplementary Figure S7. Scatterplot of crown-heel length measured with the two measurement devices for each infant at preterm age.

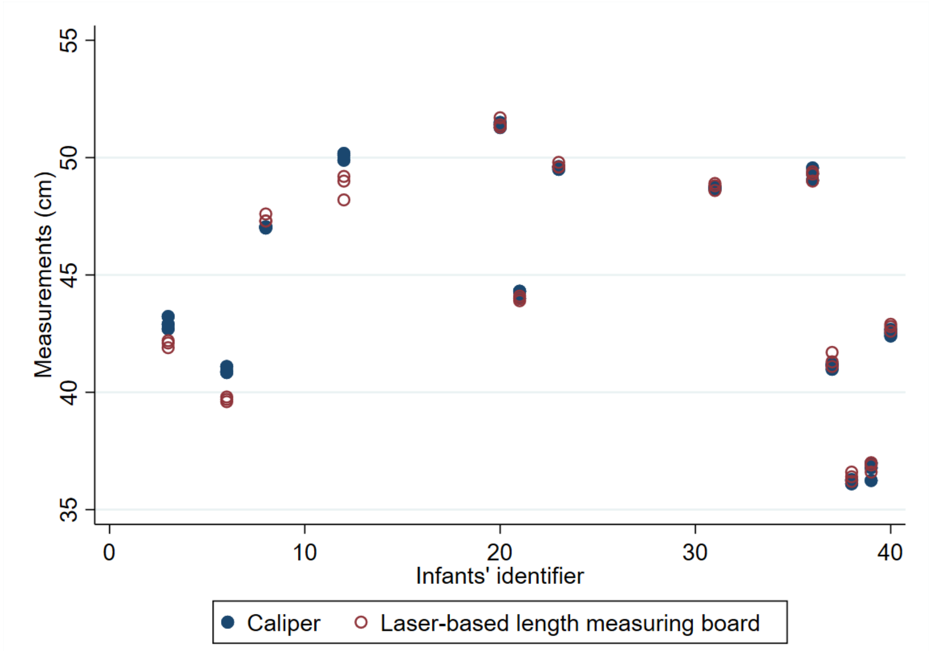

Supplementary Figure S8. Scatterplot of crown-heel length measured with the two measurement devices for each infant at term.

SUPPLEMENTARY TABLES

Supplementary Table 1. Caliper readings during the calibration measurement process at the six locations shown in Supplementary Figure 6, using a calibration standard rod of 125 mm. The reading error is 0.025 mm (half the resolution of the caliper).

Location identification (Supplementary Figure S6)	Readings on the caliper (mm)
A	125.000
B	124.900
C	124.775
D	125.050
E	124.925
F	124.800

Supplementary Table 2. Intra-observer repeatability for crown-heel length measurements in infants at preterm and term corrected gestational age.

Measurement methods	ICC	95% CI
Caliper:		
- Measurements at preterm postmenstrual age	0.998	0.994-0.999
- Measurements at term postmenstrual age	0.999	0.997-0.999
Laser-based length measuring board:		
- Measurements at preterm postmenstrual age	0.997	0.993-0.999
- Measurements at term postmenstrual age	0.998	0.995-0.999

CI: confidence interval; ICC: intraclass correlation coefficient
